# Establishing a malaria diagnostics centre of excellence in Kisumu, Kenya

**DOI:** 10.1186/1475-2875-6-79

**Published:** 2007-06-12

**Authors:** Colin Ohrt, Peter Obare, Ampon Nanakorn, Christine Adhiambo, Ken Awuondo, Wendy Prudhomme O'Meara, Shon Remich, Kurt Martin, Earnest Cook, Jean-Paul Chretien, Carmen Lucas, Joseph Osoga, Peter McEvoy, Martin Lucas Owaga, James Sande Odera, Bernhards Ogutu

**Affiliations:** 1 Walter Reed Army Institute of Research, Robert Grant Avenue, Silver Spring, Maryland, USA; 2 Malaria Diagnostics Centre of Excellence, Centre for Clinical Research, Kenya Medical Research Institute, PO Box 54, Kisumu, Kenya & Walter Reed Project, United States Army Medical Unit-Kenya, PO Box 54, Kisumu, Kenya; 3 Armed Forces Research Institute of Medical Science, Rajvithi Road, Bangkok, Thailand; 4 Fogarty International Center, National Institutes of Health, Center Drive, Bethesda, Maryland, USA; 5 Naval Medical Research Center Detachment, Av. V CDRA 36 Callo 2,Lima, Peru; 6 Armed Forces Institute of Pathology, 16th Street NW, Washington DC, USA; 7 Centers for Disease Control and Kenya Medical Research Institute, PO Box 54, Kisumu, Kenya

## Abstract

**Background:**

Malaria microscopy, while the gold standard for malaria diagnosis, has limitations. Efficacy estimates in drug and vaccine malaria trials are very sensitive to small errors in microscopy endpoints. This fact led to the establishment of a Malaria Diagnostics Centre of Excellence in Kisumu, Kenya. The primary objective was to ensure valid clinical trial and diagnostic test evaluations. Key secondary objectives were technology transfer to host countries, establishment of partnerships, and training of clinical microscopists.

**Case description:**

A twelve-day "long" and a four-day "short" training course consisting of supervised laboratory practicals, lectures, group discussions, demonstrations, and take home assignments were developed. Well characterized slides were developed and training materials iteratively improved. Objective pre- and post-course evaluations consisted of 30 slides (19 negative, 11 positive) with a density range of 50–660 parasites/μl, a written examination (65 questions), a photographic image examination (30 images of artifacts and species specific characteristics), and a parasite counting examination.

**Discussion and Evaluation:**

To date, 209 microscopists have participated from 11 countries. Seventy-seven experienced microscopists participated in the "long" courses, including 47 research microscopists. Sensitivity improved by a mean of 14% (CI 9–19%) from 77% baseline (CI 73–81 %), while specificity improved by a mean of 17% (CI 11–23%) from 76% (CI 70–82%) baseline. Twenty-three microscopists who had been selected for a four-day refresher course showed continued improvement with a mean final sensitivity of 95% (CI 91–98%) and specificity of 97% (CI 95–100%). Only 9% of those taking the pre-test in the "long" course achieved a 90% sensitivity and 95% specificity, which increased to 61% of those completing the "short" course. All measures of performance improved substantially across each of the five organization types and in each course offered.

**Conclusion:**

The data clearly illustrated that false positive and negative malaria smears are a serious problem, even with research microscopists. Training dramatically improved performance. Quality microscopy can be provided by the Centre of Excellence concept. This concept can be extended to other diagnostics of public health importance, and comprehensive disease control strategies.

## Background

Quality of laboratory medicine has always been a barrier to effective health care in the developing world. The issues and potential solutions have been recently illustrated by Petti, Bates and colleagues [1,2]. Laboratory end points are a challenge in clinical trials in this setting as well.

Malaria microscopy remains the reference or "gold" standard for malaria diagnosis in clinical trials (drug and vaccine), new diagnostic methodology evaluation, and for clinical care for much of the world today. It is known that microscopy is an imperfect gold standard [3-5]. The accuracy of microscopy relates to innate ability, training, experience, motivation and laboratory resources.

New diagnostic methods are now available, including malaria rapid diagnostic tests (RDTs) and PCR [3,5,6]; however, neither are yet validated in the clinical trial setting. RDTs are coming into widespread clinical use in some parts of the world, and they may well become key to malaria management and control in many settings. Currently available devices have limitations, such as batch-to-batch quality variation, species and density determination, persistent positivity, accuracy and cost [6]. Also of concern with RDTs are the reported false negative results in the presence of high parasitaemia [6]. The usefulness of RDTs in higher transmission areas of Africa remains to be determined, and comparative cost effectiveness studies are needed.

Sensitivity and specificity of the new diagnostic techniques have been evaluated based on microscopy. Variability in the accuracy of microscopy certainly accounts for some of the wide spectrum of reported sensitivity and specificity of these new devices [5-7]. Because of various limitations of new methods, microscopy will continue to have a significant role in the clinical trial setting for several years to come, and as the reference standard to which new diagnostic devices are compared. Expert microscopy should be available not only in reference centers, but also in clinical trial sites worldwide.

Errors with microscopy and the need for uniform training, quality assurance, and standardized reporting methods have been noted in the literature [8,9]. A recent workshop in Malaysia details the problem and outlines methods for achieving quality with malaria microscopy [10]. Accuracy improvement with training and methods modification have also been reported [2,11]. Yet, detailed methods used for microscopic endpoints in clinical trials are not the norm, nor are quality control measures often in place [7]. In addition, microscopists' proficiency is often poorly defined. Based on these issues, outcomes of several clinical trials have been in question in the two last decades (unpublished observations).

Where microscopy is available in the clinical setting, physicians often treat patients despite negative blood film reports, because of lack of confidence in the report [12]. False positive results may lead to failure to consider or delay in the treatment of alternative life-threatening diagnoses [1] and contribute to the cycle of ill health and increasing poverty [13]. Accurate diagnosis in the clinical setting is now more important than ever. Artemisinin-based combinations (ACTs) overuse may result in more rapid emergence of drug resistance. The relatively high cost of these new drugs will make accurate diagnosis likely to be cost effective [7].

This manuscript details problems, outlines pitfalls, and provides solutions for successful, validated malaria microscopy. These solutions will help provide valid results in the clinical trial setting when microscopy is used as the primary endpoint. The solutions presented are certainly not unique to microscopy. Lessons learned, solutions proposed, and the Centre of Excellence concept can be widely adapted to improve clinical trials and health care worldwide, especially in the developing world.

## Case description

### Malaria Diagnostics Centre of Excellence in Kisumu, Kenya

#### Primary mission, objectives and approach

The Malaria Diagnostics Centre of Excellence in Kisumu, Kenya was established in March 2004. The primary mission for the Centre of Excellence is to ensure valid microscopy when it is used to determine the primary endpoint in clinical trials or to assess new diagnostic methods. This mission is being accomplished through standardized and quality training, consistent methods documented in standard operating procedures (SOPs), a critically evaluated rereading paradigm (for blood film reading), quality control and assurance, and objective assessment and documentation of each microscopist's performance, in both the formal testing setting and in everyday job performance.

Additional objectives are to train microscopists working in the developing world in the clinical setting, to transfer technology to host countries, to become internationally recognized, and to establish partnerships with organizations with similar missions. Transfer of technology to host countries is occurring through mentoring and motivating host country leadership, training trainers for other locations, and transferring training materials to other centers. The intention is to become internationally recognized through training leaders, offering/advertising capability, and by conducting, presenting, and publishing operational research results in areas of need.

Seven expert microscopists from four continents (Kenya 3, Peru 1, Indonesia 1, USA 1, and Thailand 1) were identified beginning in 2001. Standardized training was developed through their experience, review of relevant training materials, and iterative improvement based on constructive feedback and training results over two years. Useful books and plates are available from WHO for purchase or download [14,15].

Well-characterized slides were developed for formal slide reading examinations. Slide examinations comprised well characterized slides with low density parasitaemia of *Plasmodium falciparum, Plasmodium malariae, Plasmodium ovale*, true negatives, and serially diluted *P. falciparum *slides (for counting). Training slide sets have been generated for each organization to have on site for on-going training. Negative slides are prepared from non-exposed people newly arriving to endemic areas to avoid the issue of the low-parasite density healthy cases common in malaria endemic areas. Fully validated species identification and counting examinations are under development.

A written examination was developed and now consists of 65 multiple choice questions and a question bank to assess microscopists' knowledge of key concepts and facts. A digital picture test evaluation was also developed to teach and evaluate microscopists' ability to correctly identify characteristics of artifact and of various growth stages of each malaria species. PowerPoint presentations were developed to train didactic and visual concepts necessary for accurate microscopy. The test questions, pictures and presentations have been iteratively improved over time.

Over the last four years, SOPs have been developed and iteratively improved at the Kombewa research site near Kisumu. These include SOP's for sample collection and reception, slide preparation, buffer preparation, stain preparation and staining of slides, slide reading, rereading paradigm, slide storage, and quality control. These SOPs are compared with the SOPs of the participants, and provided to course participants to take home.

#### Course resources, content and methodology

The venue for training is the Walter Reed Project Guesthouse lecture hall, located in Kisumu, Kenya. Laboratories at the nearby sites are used for laboratory practicals. Courses are conducted by experienced facilitators and senior microscopists. Currently, the Centre has three full-time staff (Centre director/Course director, a microscopist, and a database specialist), a part-time physician director, part-time laboratory officer, and additional three to six expert microscopists available for teaching. A twelve-day "long" course was designed for first time training, and a four-day "short" course is available as a refresher for those who performed well in the long course.

Training targets malaria microscopists (laboratory technologists, researchers) from research and clinical laboratories. Entry requirements include at least a diploma or certificate in medical laboratory technology and one year of experience as a malaria microscopist. Attendees are currently required to be proficient in written and spoken English.

Essential equipment included 23 microscopes, four computers, and two computer projectors. Fifteen high quality power point presentations with lecture notes, a slide bank of well-characterized malaria smears, a picture bank, WHO Bench Aids for the Diagnosis of Malaria [14], WHO Basic Malaria Microscopy yellow book [15], and SOPs are used for training materials. Supplies for making slides are available. Each visiting institution has been provided with WHO training plates and Basic Malaria Yellow Book [14,15], Walter Reed Project standard operating procedures, and training sets of 50 slides. The intent of providing these materials is to continue to improve skills between courses and in order to ultimately certify microscopists.

Teaching methods consist of supervised laboratory practicals, open laboratory time, lectures, group discussions/presentations, demonstrations, and take home assignments. Objective assessment include a pre- and post-training written test, positive-negative slide test, species identification slide test, counting slide test, artifact and species identification picture test, and slide preparation practical. The agenda for the "long" course is presented in Table 1. The "short" course agenda is presented in Table 2.

**Table 1:** Microscopy "long" course training agenda

**Day**	**Time**	**Topic**
1	9.00 – 9.30	Opening of the session
	9.30 – 10.30	Introduction/Motivation/Goals of the course/Trials and tribulations
	10.45 – 11.45	Clinical presentation of malaria
	11.45 – 12.45	Qualities of a good microscopist
	1.45 – 2.45	Microscope maintenance
	2.45 – 3.45	Standard operating procedure (SOP) development
	4.00 – 5.00	Multiple choice questions (MCQ) written pre-test
	Homework	Compare individual SOPs to Walter Reed Project SOPs. Note differences.
2	8.00 – 9.00	Good Clinical Laboratory Practice/Quality control
	9.00 – 10.00	Photographic image pre-test
	10.15 – 1.15	Slide reading pre-test (30 slides: 5 minutes each, 1 minute for passing the slide)
	2.00 – 4.00	Counting pre-test (12 slides: 10 minutes each)
	4.15 – 5.15	Review written test
	Homework	Review Bench Aids for the diagnosis of malaria infections
3	8.00 – 10.00	Slide preparation, review form for scoring slide quality
	10.15 – 11.15	Malaria quality assurance & control
	2.00 – 5.00	**Laboratory – Slide preparation and staining**
4	8.00 – 9.30	Species identification & groups' discussion
	9.45 – 12.45	**Laboratory – Species identification**
	1.45 – 2.45	Approach to mixed infections
	2.45 – 3.45	Significance of false positives & negatives and counting
	4.00 – 5.00	Review photographic image test with discussion
5	8.00 – 9.30	Artifacts, pseudo-parasites and the reality of false positives
	9.45 – 12.00	**Laboratory – Artifacts**
	1.00 – 3.45	**Laboratory – Slide reading, address individual difficulties**
	4.00 – 5.00	SOPs – Groups' presentations and discussions
6, 7	Weekend	**Open microscopy laboratory**
8	8.00 – 9.00	Malaria life cycle
	9.00 – 10.00	Counting basics and techniques
	10.15 – 12.00	**Laboratory – Practice counting using Walter Reed Project SOP**
	1.00 – 4.30	**Laboratory – Compare individual technique results to SOP technique results**
	4.45 – 5.15	Review day 1 to 5 microscopy
9	8.00 – 9.00	Low density infections
	9.00 – 9.30	Cross contamination experiment
	9.45 – 5.30	**Laboratory – Individual laboratory in reference to the pretest scores**
10	8.00 – 9.00	Malaria rereading paradigm
	9.00 – 10.00	Review of the lectures
	10.15 – 12.30	Questions and trouble points, Panel discussion
	1.30 – 5.00	**Laboratory – Review and scoring quality of blood slides**
11	8.00 – 10.30	Slide reading post-test
	10.45 – 11.45	Written post-test
	11.00 – 12.15	Photographic image post-test
	1.00 – 3.00	Counting post-test
	3.00 – 5.00	**Laboratory – Open microscopy and individual help**
12	8.00 – 2.00	Go over tests and results, give out take-home training materials

**Table 2:** Microscopy "short" course training agenda

**Day**	**Time**	**Topic**
1	8.00 – 9.00	Opening of the session
		Introduction to malaria microscopy certification
	9.00 – 11.15	Slide reading pre-test (30 slides: 5 minutes each, 1 minute for passing the slide)
	11.30 – 12.30	Written pre-test
		Species identification pre-test (20 slides: 5 minutes each, 1 minute for passing the
	1.00 – 4.00	slide)
	4.15 – 6.15	Counting pre-test (12 slides: 10 minutes each)
2	7.30 – 8.00	Review written pre-test
	8.00 – 11.15	**Laboratory – Review slide reading pre-test with discussion**
	11.30 – 12.15	**Laboratory – Review species identification pre-test with discussion**
	1.00 – 4.00	**Laboratory – Review counting pre-test with discussion**
	4.15 – 6.15	**Laboratory – Individual laboratory help**
3	7.30 – 8.00	Artifacts
	8.00 – 9.45	**Laboratory – Artifacts**
	10.00 – 10.30	Species identification
	10.30 – 12.45	**Laboratory – Species identification**
	1.30 – 2.00	Low density infections
	2.00 – 4.00	**Laboratory – Low density infections**
	4.15 – 4.45	Significance of false positive and negative results
	4.45 – 6.30	**Laboratory – Individual laboratory help, address difficulties**
4	7.30 – 8.15	Written post-test
	8.15 – 11.15	Slide reading post-test
	11.30 – 1.15	Species identification post-test
	1.45 – 4.45	Counting post-test
	5.00 – 6.00	Awarding of certificates

#### Objective evaluations

A written test comprising 65 questions was given as a pre- and post-test. The following areas were covered: Good Clinical Laboratory Practice/standard operating procedures (32% of test questions), malaria parasite morphology/stages (31%), slide preparation and staining (14%), microscopy results interpretation (11%), microscope care/knowledge (5%), general malaria knowledge (3%), sensitivity/specificity calculation (2%), and density determination (2%). A practical slide reading examination of 30 slides was given before and after the course. Nineteen slides were true negative, 11 were positive, and of the positive slides, nine were *P. falciparum*, one was *P. ovale*, and one was *P. malariae*. The density range of the positives was 50–660 parasites/μl, with a mean of 220 and median of 142. Of the positive slides, one was 50 parasites/μl, five were between 100–200 parasites/μl, and five were between 201–660 parasites/μl.

Negative slides were collected in Jakarta and Bangkok from healthy individuals in malaria free areas, who had not traveled to malaria endemic areas or taken malaria prophylaxis in the past two years or had malaria in the last 10 years. In Kenya, slides were collected from newly arrived previously non-exposed individuals within five days of arrival to Kenya. This was to ensure that the donors were truly negative. Thirty-two percent of all true negative smears were spiked with artifact (a mix of *Staphylococcus aureus *and fungi) at varying densities (1/100 high power fields -2 per high power field). Negatives were validated as negative by sampling criteria and by giving each of the 209 participants the opportunity to challenge the results with the Centre experts.

Sixty-six percent of positive smears were from the Malaria Research and Reference Reagent Resource Center (MR4) collection effort in Cambodia and contained *P. falciparum *(not part of the available sets). MR4 positives were determined to be positive and species identified by two expert readers in Indonesia with confirmation by three independent expert readers at the Centre. These were supplemented by slides collected in Kenya containing *P. falciparum*, *P. malariae*, and *P. ovale*. These were independently confirmed positive by one expert from Thailand and three experts at the Centre. Positive smears were further validated by giving each of the 209 participants the opportunity to challenge the results with the Centre experts (asking staff to identify the parasites for them).

The slides used in practical slide reading examinations were identical, but renumbered in the post-examination. Participants were given five minutes per slide for reading. One minute was provided between slides. They were instructed to read 200 high power fields before calling the smear negative. The presence or absence of parasites and the species was recorded.

Ability to properly identify species and artifact was assessed in a photographic image examination. The images included 11 without and 19 with malaria parasites. Of those without parasites, various artifacts were present. Images with parasites were distributed as follows: five *P. falciparum*, four *P. malariae*, five *P. ovale*, two *Plasmodium vivax*, and three mixed species with various stages present.

A slide preparation practical emphasized correct sample collection methods for making malaria blood smears, smear preparation, preparation of stains, and individual slide staining. Participants were then expected to make smears using their own SOPs and the Walter Reed Project SOPs. Five smears from each SOP were then assessed on a standardized form, and the participant assigned a passing or failing grade.

#### Interpretation of results, data management, and analysis

Written examination questions were scored as one point each. Practical slide reading examinations were reported as sensitivity, specificity, and species error. Sensitivity was calculated as Sensitivity = (True Positives/[True Positives + False Negatives]). Specificity was calculated as Specificity = (True Negatives/[True Negatives + False Positives]). Data were entered into, verified in, and calculations performed with Microsoft Office Excel 2003. Further statistical analyses were performed and tables constructed with SPSS 14.0 for Windows. Pre- and post-test means were compared with paired sample T-tests. Means were compared with ANOVA, and proportions with Fisher's Exact Test (2-sided). P values were not corrected for multiple comparisons.

## Results

To date, 209 microscopists have participated from the following countries: Kenya (178), Uganda (10), Tanzania (5), Rwanda (1), Burundi (1), Malawi (6), Cameroon (3), Mali (2), Nigeria (2), and Thailand (1). One hundred and fifty eight participants (76%) have been primarily research and 51 (24%) have been primarily clinical. Leadership/expertise has participated from Kenya (4), Uganda (1), Nigeria (2), Mali (1), Cameroon (1), Zanzibar (2), Thailand (1), Peru (1), USA (1), and Indonesia (1).

Data from the courses between July 2005 and January 2006 are reported here. These data include 77 participants in the long course (including 10 who trained twice) and 23 participants in the short course (including five who trained twice). Of these, 69% were conducting primarily research. Participants were from Kenya (81), Tanzania (5), Malawi (4), Rwanda (1), Burundi (1), Cameroon (2), Nigeria (2), Mali (2), and Uganda (2). On a questionnaire, participants reported a mean (median) of years of laboratory education 3 (3), years employed as a laboratory technician or technologist 9 (6), and years employed as a malaria microscopist 5 (3). Twelve percent reported that they were laboratory supervisors, 30% reported that they had a malaria proficiency program in their laboratory, and 84% reported that they used SOPs in their laboratory.

Pre- and post-training scores and percent improvement by organizational group and course type from the most recent participants are presented in Additional File 1. With a mean specificity of 80% and range in mean by organization on the pre-test of 66–91%, these data clearly illustrate this is a serious problem in both the research and clinical setting. Specificity improved substantially with training (Additional File 1).

Thirty-two percent of all true negative smears were spiked with artifact (mix *Staphylococcus aureus *with fungi at varying densities). On the pre-test, 26% of those spiked with artifact were read as false positives, while 18% of those not spiked were. On the post-test, 8% of those spiked with artifact were read as false positives, while 6% of those not spiked were. The slides with the highest density artifact had higher rates of false positives. Spiked slides did not appear to affect the species reported, except for a slightly higher rate reported as *P. vivax *in the post-test. The false positive smears reported as *P. vivax *and *P. ovale *increased overall in comparing the pre-test with the post-test, while the reporting of *P. falciparum *decreased.

The effect of parasitaemia on the false negative rate was not as large as anticipated. The mean (median) densities of those with false negatives on the pre-test were 174 (128) parasites/μl compared with 216 (142) parasites/μl for the true positives. The densities were 133 (120) and 216 (142) parasites/μl for the false negatives and true positives on the post-test, respectively.

Short course participants were selected from those who previously scored well in a long course. Comparison between the long and short course scores in Additional File 1 suggests that improved scores were maintained from prior training, and that improvement continued with additional training (Additional File 1).

The percentage of participants achieving sensitivity and specificity cut points on final examination by organization and course type is presented in Additional File 2. Kenya research organizations have emphasized quality microscopy for a number of years. This is reflected in the percentage of individuals able to achieve acceptably high cut points. For both research and clinical organizations, this approach can select those qualified to be employed as a microscopist and a given level of expertise. This approach will be used to determine cut points for certification at three levels of expertise.

CoE microscopy training appeared to substantially contribute to obtaining a level of 90% sensitivity and specificity on the pre-test, post-test, or both. Those that passed neither had a mean (sd) of 0.14 (0.50) courses attended previously, while those that passed just the post-test had a mean of 0.5 (1.0) courses attended previously, and those that passed both had attended 1.1 (1.6) course previously (p = 0.004). Use of SOPs, having a malaria proficiency program in the laboratory, and number of Good Clinical Practice trials conducted appear to correlate with ability to pass at a 90% sensitivity and specificity level (p = 0.014, 0.014, and p < 0.001, respectively). Years of training, years of experience, being a laboratory supervisor, and Good Clinical Practice courses did not appear to affect ability to score well.

## Discussion and evaluation

These data clearly illustrate that malaria microscopy is problematic. The ultimate goal should be its replacement by affordable, more robust diagnostic techniques, where valid microscopy is not achievable. The Centre will help ensure that microscopy is providing a valid "gold standard" comparator as these new tools continue to become available and need evaluation for regulatory approval and operational assessment. As long as microscopy is used to determine the primary endpoint in malaria prevention and treatment trials, great lengths must be taken to ensure that good products are not inadvertently discarded. A 1% false positive rate with serially collected smears can under estimate protective efficacy by 10–30% [8].

Many of the participants describe the training as a life changing experience. They report they did not know they were not accurately diagnosing malaria. After attaining good test scores, they say they can now work with confidence. They report it is not just malaria diagnosis that will be improved, but all laboratory procedures they are involved in. Certification is strongly desired.

The results are encouraging, but it remains to be seen if improved results in the classroom setting translate to the field, and if this concept is sustainable. Certification criteria are currently being developed along with a plan to certify microscopists at a basic level from a limited number of institutions in Kenya in the next twelve months. The plan is to also offer an advanced level and expert level of certification in the near future. Certificates will be valid for two years. For a broader impact, the plan is to train trainers from other Centres to enable development of other Centres of Excellence.

Government sector laboratory technicians are having more difficulty achieving acceptable scores in the long training course (Additional File 1). A modified and lengthened course specific to their needs may be needed. Changes in the policy and methods employed in this setting are also likely necessary. RDTs are a logical alternative. However, RDT accuracy in the clinical setting of holoendemic Africa must be determined. The simple presence or absence of parasitaemia may not distinguish severe disease from malaria. The microscope will continue to be needed for a variety of other diagnoses in the clinical setting [16,17]. Accuracy and cost effectiveness of simplified malaria diagnosis (e.g. routine use of the thin smear) should be assessed.

Errors with microscopy include false positives (specificity), false negatives (sensitivity), species error (wrong species or missed mixed infections), and counting error [8,9,18-22]. Although not widely recognized, false positive smears are common with average microscopists (Additional File 1). In the clinical setting, false positive malaria smears may lead to no or delayed treatment for the actual underlying disease, and therefore may also be a cause of mortality [1,20]. In the research setting, false positive smears will lead to underestimated protective efficacy in malaria prevention trials [8] and efficacy in treatment trials. This may result in inappropriately categorizing a vaccine or drug as not worth developing. It is suspected that efficacy may have been underestimated in a variety of trials conducted in the last 15 years. This series of suspicions culminated in a trial where mefloquine's estimate of "prophylactic efficacy" as a positive control in a Phase III clinical trial was 1% (unpublished data). An investigation revealed false positive malaria smears to be the cause. In studies where a positive control is not employed (e.g. malaria vaccine trials), such a problem may go completely undetected.

Prestudy assessment of microscopists using a very limited number of slides may help, but will not completely solve the problem. For example, using blinded assessments of a well-validated 15 slide test set, qualified microscopists were identified for a recent malaria diagnostic pivotal trial. Subsequently, internal QC mechanisms determined that one senior microscopist still made errors in excess of 10%, requiring re-interpretation of hundreds of slides and a substantial delay in completion of the analysis (RA Gasser, personal communication).

False negative smears are expected at low parasite densities (e.g. < 100 parasites/μl) by chance [23]. Insufficient reading time, poor smear quality, lack of motivation, and poor equipment will also lead to false negative smears. False negative smears will result in a lower than expected attack rate if the study population is semi-immune and does not become ill with low parasitaemia, but do not adversely impact protective efficacy estimates in non-immune or semi-immune populations (unless there is an interaction between the test agent and immunity in semi-immunes) [8]. False negative smears may lead to delayed diagnosis and severe illness in non-immune study subjects. This also results in clinicians ignoring the laboratory results in malaria endemic areas [12].

Species errors are common with low density infections, especially when only ring forms are seen [8,21]. Missed mixed infections appear to be exceedingly common when microscopy is compared with PCR [8,21,22]. Species errors could have the effect of false positive or negative smears if a species-specific endpoint is defined (e.g. malaria vaccines).

Substantial variability in counting results is a common problem. Counting errors may impact clinical trials if parasite density is an endpoint (e.g. malaria vaccines, early malaria treatment failures). O'Meara and colleagues have explored the factors affecting variability and assessed a grid method for improving accuracy [24].

Several factors will lead to improved endpoints and confidence in results in both the clinical trial and clinical setting. Suggestions are as follows for clinical trial setting: proficiency training and testing leading to certification of microscopists at varying levels of expertise, a rereading paradigm specifically designed for the situation, SOPs, quality control and assurance, laboratory standards, and reporting guidelines.

## Abbreviations

RDT: Rapid Diagnostic Test, QA/QC: Quality Assurance/Quality Control, ACTs: Artemisinin-based combination therapy, MR4: Malaria Research and Reference Reagent Resource Center, NIH: National Institutes of Health, SOP: Standard Operating Procedure, WHO: World Health Organization, CoE: Centre of Excellence, GCLP: Good Clinical Laboratory Practices

## Authors' contributions

CO served as the Principle Investigator for funding and execution of project; led evaluation of a problematic clinical trial, conceived the Malaria Diagnostics Centre of Excellence concept, assisted with CoE design, teaching, data collection, analysis, interpretation of data and wrote this manuscript. AN was the lead visiting expert microscopist, and assisted with CoE design, teaching, data collection, analysis, interpretation of data. PO is lead microscopist/laboratory technician at the CoE who assisted with CoE design, teaching, data collection, analysis, interpretation of data. CA is lead administrator of CoE, assisted with CoE design, teaching, data collection, analysis, interpretation of data. KA was a key facilitator of the CoE during its inception, led CoE design, assisted with teaching, data collection, analysis, interpretation of data. WPO was a visiting scientist to the CoE, assisted with teaching, conception and design of data capture, revised manuscript critically for substantial intellectual content. SR was responsible for day-to-day management of CoE, assisted with CoE design, teaching, and data collection. KM was laboratory supervisor, Western Kenya; assisted with day-to-day management of CoE; teaching, and data collection. JC analyzed data; and revised manuscript critically for substantial intellectual content; EC assisted with CoE design, teaching, and interpretation of the data. CL was a lead visiting expert microscopist, and assisted with CoE design, teaching, data collection, analysis, and interpretation of data. JO was a CoE microscopist, who assisted with CoE design, teaching, and data collection. PM was a visiting microscopist who assisted with CoE design, teaching, data collection, analysis, interpretation of data. JSO and MLO were the Kenyan expert microscopists who interpreted all smears, proctored all laboratories, and were critical to developing all training materials. BO is Director of the CoE who led CoE design, teaching, data collection, analysis, interpretation of data. All authors read and approved the manuscript.

## Supplementary Material

Additional file 1 Pre-test scores and percentage improvement following training for each examination Click here for file

Additional file 2 Percentage of participants achieving sensitivity and specificity cut points on initial examination Percentage of participants achieving sensitivity and specificity cut points on final examination Improvement of participants achieving sensitivity and specificity cut points on initial examination Click here for file
