# Supplementary material for: Establishing a malaria diagnostics centre of excellence in Kisumu, Kenya
Source: Malar J. 2007 Jun 12;6:79. doi: 10.1186/1475-2875-6-79 (PMC1933544; doi:10.1186/1475-2875-6-79)
Supplement: Additional file 1 — Pre-test scores and percentage improvement following training for each examination [file 1475-2875-6-79-S1.pdf]

Additional File 1. Pre-test scores and percentage improvement following training for each examination

Group	N	*Written Examination* Pre-test	*Written Examination* Improvement	*Picture Score* Pre-test	*Picture Score* Improvement	*Sensitivity* Pre-test	*Sensitivity* Improvement	*Specificity* Pre-test	*Specificity* Improvement
Kenya Research Organizations	55	74% (69-78)*	*20%* (17-23)	57% (53-61)	*21%* (18-25)	82% (76-88)	*10%* (5-15)	84% (78-91)	*12%* (6-18)
Other Country Research Org	14	63% (55-72)	*25%* (20-30)	56% (49-62)	*24%* (16-33)	66% (53-79)	*24%* (12-36)	81% (66-96)	*9%* (-7-26)
Malaria Control Program	6	50% (34-65)	*31%* (17-44)	53% (44-63)	*12%* (3-22)	80% (62-99)	*8%* (-7-22)	91% (83-100)	*4%* (.2-9)
Teaching Staff	2	65% (26-104)	*23%* (NA)	56% (-43-154)	*26%* (1-51)	61% (-205-327)	*12%* (-23-47)	68% (2-135)	*24%* (-10-57)
Private Clinics	11	57% (48-66)	*30%* (22-37)	51% (43-59)	*25%* (15-35)	78% (67-88)	*7%* (-9-23)	66% (43-89)	*29%* (7-51)
Government Clinics	12	54% (49-59)	*35%* (30-40)	46% (34-57)	*29%* (13-45)	67% (53-82)	*14%* (-1-29)	67% (55-79)	*16%* (3-30)
Total/Mean	100	66% (63-70)	*24%*** (22-27)	55% (52-57)	*23%*** (20-26)	77% (73-81)	*12%*** (8-16)	80% (75-85)	*14%*** (9-18)
Long Course	77	62% (58-65)	*27%*** (25-30)	54% (51-57)	*24%*** (21-27)	74% (68-79)	*14%*** (9-19)	76% (70-82)	*17%*** (11-23)
Short Course	23	82% (78-87)	*15%*** (11-18)	57% (52-62)	*18%*** (11-24)	89% (83-95)	*6%* (-1-12)	94% (90-98)	*3%* (-.5-7)

* 95% confidence interval

** p<0.001, comparisons of only total, long, and short pre and post examination means
