# Supplementary material for: Establishing a malaria diagnostics centre of excellence in Kisumu, Kenya
Source: Malar J. 2007 Jun 12;6:79. doi: 10.1186/1475-2875-6-79 (PMC1933544; doi:10.1186/1475-2875-6-79)
Supplement: Additional file 2 — Percentage of participants achieving sensitivity and specificity cut points on initial examination Percentage of participants achieving sensitivity and specificity cut points on final examination Improvement of participants achieving sensitivity and specificity cut points on initial examination [file 1475-2875-6-79-S2.pdf]

Additional File 2.

Percentage of participants achieving sensitivity and specificity cut points on initial examination

	n	*Senstivity/Specificity* 80/80	80/85	80/90	80/95	85/90	85/95	85/97.5	85/100	90/90	90/95	90/97.5	95/97.5
Kenya Research Organizations	55	55%	47%	40%	29%	31%	20%	20%	20%	31%	20%	20%	13%
Other Country Research Org	14	21%	14%	14%	7%	14%	7%	7%	7%	14%	7%	7%	0%
Malaria Control Program	6	67%	67%	33%	33%	17%	17%	17%	17%	17%	17%	17%	0%
Teaching Staff	2	0%	0%	0%	0%	0%	0%	0%	0%	0%	0%	0%	0%
Private Clinics/Hospitals	11	18%	18%	9%	0%	9%	0%	0%	0%	9%	0%	0%	0%
Government Clinics/Hospitals	12	25%	8%	0%	0%	0%	0%	0%	0%	0%	0%	0%	0%
Total	100	42%	35%	27%	19%	21%	13%	13%	13%	21%	13%	13%	7%
Long Course	77	32%	25%	18%	13%	14%	9%	9%	9%	14%	9%	9%	4%
Short Course	23	74%	70%	57%	39%	43%	26%	26%	26%	43%	26%	26%	17%

Percentage of participants achieving sensitivity and specificity cut points on final examination

	n	*Senstivity/Specificity* 80/80	80/85	80/90	80/95	85/90	85/95	85/97.5	85/100	90/90	90/95	90/97.5	95/97.5
Kenya Research Organizations	55	85%	84%	78%	60%	67%	51%	51%	51%	67%	51%	51%	29%
Other Country Research Org	14	64%	57%	57%	36%	50%	29%	29%	29%	50%	29%	29%	14%
Malaria Control Program	6	83%	83%	83%	50%	67%	33%	33%	33%	67%	33%	33%	17%
Teaching Staff	2	50%	50%	0%	0%	0%	0%	0%	0%	0%	0%	0%	0%
Private Clinics/Hospitals	11	73%	55%	55%	27%	55%	27%	27%	27%	55%	27%	27%	9%
Government Clinics/Hospitals	12	50%	42%	25%	25%	17%	17%	17%	17%	17%	17%	17%	0%
Total	100	75%	70%	64%	47%	55%	39%	39%	39%	55%	39%	39%	20%
Long Course	77	71%	65%	58%	43%	48%	32%	32%	32%	48%	32%	32%	16%
Short Course	23	91%	91%	87%	61%	83%	61%	61%	61%	83%	61%	61%	35%

Percent improvement of participants in achieving sensitivity and specificity cut points

	n	*Senstivity/Specificity* 80/80	80/85	80/90	80/95	85/90	85/95	85/97.5	85/100	90/90	90/95	90/97.5	95/97.5
Kenya Research Organizations	55	31%	36%	38%	31%	36%	31%	31%	31%	36%	31%	31%	16%
Other Country Research Org	14	43%	43%	43%	29%	36%	21%	21%	21%	36%	21%	21%	14%
Malaria Control Program	6	17%	17%	50%	17%	50%	17%	17%	17%	50%	17%	17%	17%
Teaching Staff	2	50%	50%	0%	0%	0%	0%	0%	0%	0%	0%	0%	0%
Private Clinics/Hospitals	11	55%	36%	45%	27%	45%	27%	27%	27%	45%	27%	27%	9%
Government Clinics/Hospitals	12	25%	33%	25%	25%	17%	17%	17%	17%	17%	17%	17%	0%
Total	100	33%	35%	37%	28%	34%	26%	26%	26%	34%	26%	26%	13%
Long Course	77	39%	40%	40%	30%	34%	23%	23%	23%	34%	23%	23%	12%
Short Course	23	17%	22%	30%	22%	39%	35%	35%	35%	39%	35%	35%	17%
